# Dental Students' Knowledge, Attitudes and Perceptions of Artificial Intelligence Tools to Aid in the Diagnosis of Oral Cancer and Oral Potentially Malignant Disorders

**DOI:** 10.1111/odi.70225

**Published:** 2026-02-15

**Authors:** Vlaho Brailo, Rosa María Lopez‐Pintor, Molly Harte, Ana Andabak Rogulj, Danica Vidović Juras, Ivana Škrinjar, Marcio Diniz Freitas, Jacobo Limeres Posse, Luis Monteiro, Luis M. Silva, Jean‐Cristophe Fricain, Adrien Naveau, Giovanni Lodi, Niccolò Lombardi, Elena Varoni, José López López, Sonia Egido Moreno, Raj Ariyaratnam, Ali Abdullah Alqarni, Richeal Ní Ríordáin, Owen Addison, Yunpeng Li, Faleh Tamimi, Rui Albuquerque

**Affiliations:** ^1^ University of Zagreb, School of Dental Medicine, Clinic for Dentistry, University Clinical Hospital Centre Zagreb Croatia; ^2^ ORALMED Research Group, Department of Dental Clinical Specialties School of Dentistry, Complutense University of Madrid Madrid Spain; ^3^ Oral Medicine, Guy's & St Thomas' NHS Foundation Trust, Guy's Hospital London UK; ^4^ School of Medicine and Dentistry, University of Santiago de Compostela Santiago de Compostela Spain; ^5^ UNIPRO—Oral Pathology and Rehabilitation Research Unit, University Institute of Health Sciences (IUCS), CESPU Gandra Portugal; ^6^ Dental Faculty, Bordeaux University, Bordeaux University Hospital Center Bordeaux France; ^7^ INSERM U1026 BioTis Tissue Bioengineering Bordeaux France; ^8^ Dipartimento di Scienze Biomediche, Chirurgiche e Odontoiatriche Università degli Studi di Milano Milan Italy; ^9^ Department of Odontostomatology Faculty of Medicine and Health Sciences (Dentistry), University of Barcelona Barcelona Spain; ^10^ Medical Surgical Area, Dental Hospital University Barcelona (HOUB) Barcelona Spain; ^11^ Oral Health and Masticatory System Research Group Bellvitge Biomedical Research Institute (IDIBELL) Barcelona Spain; ^12^ University of Manchester Manchester UK; ^13^ Department of Oral & Maxillofacial Surgery and Diagnostic Sciences Faculty of Dentistry, Taif University Taif Saudi Arabia; ^14^ Cork University Dental School and Hospital, College of Medicine and Health, University College Cork Cork Ireland; ^15^ Centre for Oral, Clinical & Translational Sciences, Faculty of Dentistry, Oral & Craniofacial Sciences, King's College London London UK; ^16^ College Faculty of Dental Medicine and Oral Health Sciences, McGill University Montreal Canada; ^17^ Faculty of Dentistry, Oral & Craniofacial Sciences, King's College London London UK

**Keywords:** artificial intelligence, dental students, oral cancer, oral potentially malignant disorders

## Abstract

**Background:**

Artificial intelligence (AI) has emerged as a promising tool in dentistry, particularly in the early detection of oral cancer (OC) and oral potentially malignant disorders (OPMDs). Data focused on European dental students and their knowledge/attitudes towards the use of AI for diagnosing OC/OPMDs are limited.

**Methods:**

A cross‐sectional online survey was conducted among final‐year dental students from six European countries. The questionnaire assessed knowledge, attitudes, and perceived barriers regarding AI in the diagnosis of OC and OPMDs. Data were analysed using descriptive and comparative statistics to evaluate differences between countries.

**Results:**

A total of 328 students participated (75% female, 25% male). Most students stated that they had not received formal training in AI (61%) and emphasised the need to incorporate such training into the dental curriculum (47%). Students from Portugal had highest overall knowledge scores, but at the same time, lowest overall attitude scores. The most commonly perceived barrier was insufficient training (87.8%).

**Conclusion:**

Final‐year dental students demonstrate positive attitudes towards AI aiding in diagnosis of OC and OPMDs, although significant knowledge and skill gaps remain. Integrating structured AI education into dental curricula is essential to prepare future academics and clinicians for responsible and effective AI use.

## 1 Introduction

Artificial intelligence (AI) has rapidly emerged as a transformative technology across healthcare settings, including dentistry. Its capacity to analyse complex datasets, generate diagnostic predictions, and support clinical decision‐making is well documented (Schwendicke et al. 2020; Chen et al. 2024). In oral medicine specifically, AI‐based algorithms have demonstrated strong potential in detecting oral cancer (OC) and oral potentially malignant disorders (OPMDs) (Vinay et al. 2025; Karuppan Perumal et al. 2025; Veeraraghavan et al. 2024; Kouketsu et al. 2024; Redondo et al. 2026).

Oral lesions often present with a variety of clinical features, contributing to significant inter‐observer variability in diagnosis (Veeraraghavan et al. 2024; Kouketsu et al. 2024). Early detection of oral cancer depends on recognising subtle changes in tissue colour, texture, and architecture (Veeraraghavan et al. 2024; Kouketsu et al. 2024), skills that are difficult to teach using traditional methods alone (Schwendicke et al. 2020; Chen et al. 2024; Veeraraghavan et al. 2024; Kouketsu et al. 2024). AI tools can help bridge this gap by allowing students to assess clinical cases and receive immediate, objective feedback (Kouketsu et al. 2024; Redondo et al. 2026). This self‐directed correction loop accelerates learning and helps calibrate students' diagnostic “eye” to expert‐level standards (Chen et al. 2024; Kouketsu et al. 2024).

AI can also help standardise the diagnostic process (Vinay et al. 2025; Karuppan Perumal et al. 2025). By providing objective, data‐driven assessments, it reduces cognitive load and fatigue for clinicians while minimising the risk of missed detections (Veeraraghavan et al. 2024; Kouketsu et al. 2024). By enhancing diagnostic precision and decreasing observer variability, AI has the potential to support earlier identification of malignant and potentially malignant lesions, ultimately improving patient outcomes (Kouketsu et al. 2024; Redondo et al. 2026).

Given that future dental professionals are often the first to encounter patients with OC and OPMDs, the integration of AI into diagnostic workflows is particularly relevant for their training and clinical practice (Kerr et al. 2020; Coppola et al. 2021). However, for AI to be responsibly implemented in clinical practice, future dentists must not only understand its capabilities and limitations but also develop critical insight into the ethical and legal aspects of its use (Harte et al. 2025). Current exposure to AI in dental education is inconsistent and frequently theoretical (Harte et al. 2025). Most curricula provide very minimal formal teaching on the fundamentals of AI or data ethics (Thurzo et al. 2023; El‐Hakim et al. 2025). Given the growing role of AI in oral diagnostics, there is a need to assess how well dental schools prepare students for the implementation of AI in routine clinical work (Harte et al. 2025). Integrating AI into the dental curriculum is vital for equipping future dentists with the necessary skills and competency to combat the rising burden of OPMDs and OC (Coppola et al. 2021; Harte et al. 2025). Understanding students' baseline knowledge, attitudes and perceived barriers to the use of AI is essential to modify curriculum design and develop targeted educational interventions (Harte et al. 2025). Previous studies among dental students suggest that, although attitudes towards AI are generally positive, knowledge remains insufficient, highlighting the need for practical AI education in undergraduate programmes (Amiri et al. 2024; Aldakhil et al. 2024). To our knowledge, no study has examined this topic among dental students in Europe. Variations in curricular content, digital infrastructure, and clinical exposure among European dental programmes may influence students' readiness to apply AI tools in the diagnosis of OC and OPMDs, highlighting the importance of addressing this topic.

This study aimed to assess the knowledge, attitudes and perceived barriers regarding the use of AI‐based tools to aid in the diagnosis of OC and OPMDs among final‐year dental students from six European countries. Secondary objectives included evaluating how the gender and country of origin of the students influenced their responses, and the correlation between knowledge of and attitude towards the use of AI.

## 2 Materials and Methods

Ethical approval for the study was granted by the Ethics committees of all universities participating in the study. The participants consisted of final‐year undergraduate dental students from eight universities across six European countries: King's College London (United Kingdom), CESPU University (Portugal), University of Bordeaux (France), University of Milan (Italy), Complutense University of Madrid (Spain), University of Santiago de Compostela (Spain), University of Barcelona (Spain), and University of Zagreb (Croatia). The questionnaire was created using survey software (Google Forms) and disseminated to participants via email on 1st March 2025. A total of three email reminders were sent at fortnightly intervals following the initial distribution of the questionnaire. The data collection period ended on 30th June 2025. The questionnaire was anonymous, collecting only gender and country as demographic information. Participants were required to provide consent and confirm their understanding of the study's objectives before completing the questionnaire. Participation in the study was voluntary.

In the absence of a validated questionnaire for this subject area, the questionnaire was developed specifically for this study. The questionnaire took into account previously reported questionnaires from other similar studies exploring similar topics, including early detection of oral cancer and oral potentially malignant disorders, clinician knowledge and attitudes, and the role of artificial intelligence in dental education and practice (Khanagar et al. 2021; Singh et al. 2023; Aboalshamat 2022; Asmatahasin et al. 2021). The questionnaire was developed through multiple iterations until a consensus among the authors was achieved. As well as the authors, a total of twelve final‐year students from University College Cork participated in the process of questionnaire development: each student received a draft version of the questionnaire and was asked to provide feedback on its clarity and comprehensibility. All comments were collected and forwarded to the questionnaire development team for review and incorporation into the final version of the questionnaire.

The final questionnaire (Appendix A) comprised four sections. In the first section, demographic data (sex and country) were collected. The second and third sections of the questionnaire used a 5‐point Likert scale (1—strongly disagree to 5—strongly agree) to evaluate students' self‐perceived knowledge of and attitudes towards the use of AI as a diagnostic aid for OC and OPMDs. The fourth section of the questionnaire evaluated perceived obstacles to the implementation of AI in diagnosing OPMDs and OC using closed questions (yes/no).

For both the knowledge and attitude domains of the questionnaire, a total score for every participant was obtained by summing the numerical values of their responses. To ensure consistency, negatively phrased items (those indicating lower knowledge or less professional attitude) were reverse‐coded. This process reassigns numerical values to items worded in the opposite direction of the main construct, ensuring that all items uniformly represent either a high or low level of that construct. Additionally, reverse coding serves to reduce acquiescence bias (DeVellis 2013). All reversely coded statements are marked with the symbol ‘§’ in Tables 2 and 3.

Data were organised in a Microsoft Excel worksheet and stored in a shared online folder (Google Drive). Statistical analysis was conducted using SPSS version 11. The Kolmogorov–Smirnov test was employed to evaluate the normality of data distribution. Non‐parametric methods were used due to the non‐normal distribution of the data. Categorical variables were presented as proportions, while continuous variables were reported as median and interquartile range (IQR). Associations among categorical variables were evaluated using the chi‐square test, while differences among continuous variables were assessed with the Kruskal‐Wallis or Mann–Whitney test, as applicable. Spearman's correlation was used to evaluate the correlation between two continuous variables. A *p*‐value less than 0.05 (*p* < 0.05) was considered statistically significant.

## 3 Results

Three hundred twenty‐eight students (*n* = 246, 75% females; *n* = 83, 25% males) took part in the study. Total response rate was 42.7%, ranging from 11.8% to 80.4%, depending on the country. The number of participants and corresponding response rates for each country are presented in Table 1. No significant differences in the gender distribution were observed across countries.

**TABLE 1:** Number of participants and response rates by country.

	Participants (*n*, % response rate)	Female *n* (%)	Male *n* (%)	Difference in gender *p*
United Kingdom	18 (11.8%)	13 (72.2)	5 (27.8)	0.126
Portugal	76 (49.7)	59 (77.6)	17 (22.4)	
France	69 (69.7)	43 (62.3)	26 (37.7)	
Italy	45 (80.4)	36 (80)	9 (20)	
Spain	83 (38.2)	64 (77.1)	19 (22.9)	
Croatia	37 (41)	31 (83.8)	6 (16.2)	
Total	328 (42.7)	246 (75)	83 (25)	

**FIGURE 1:**
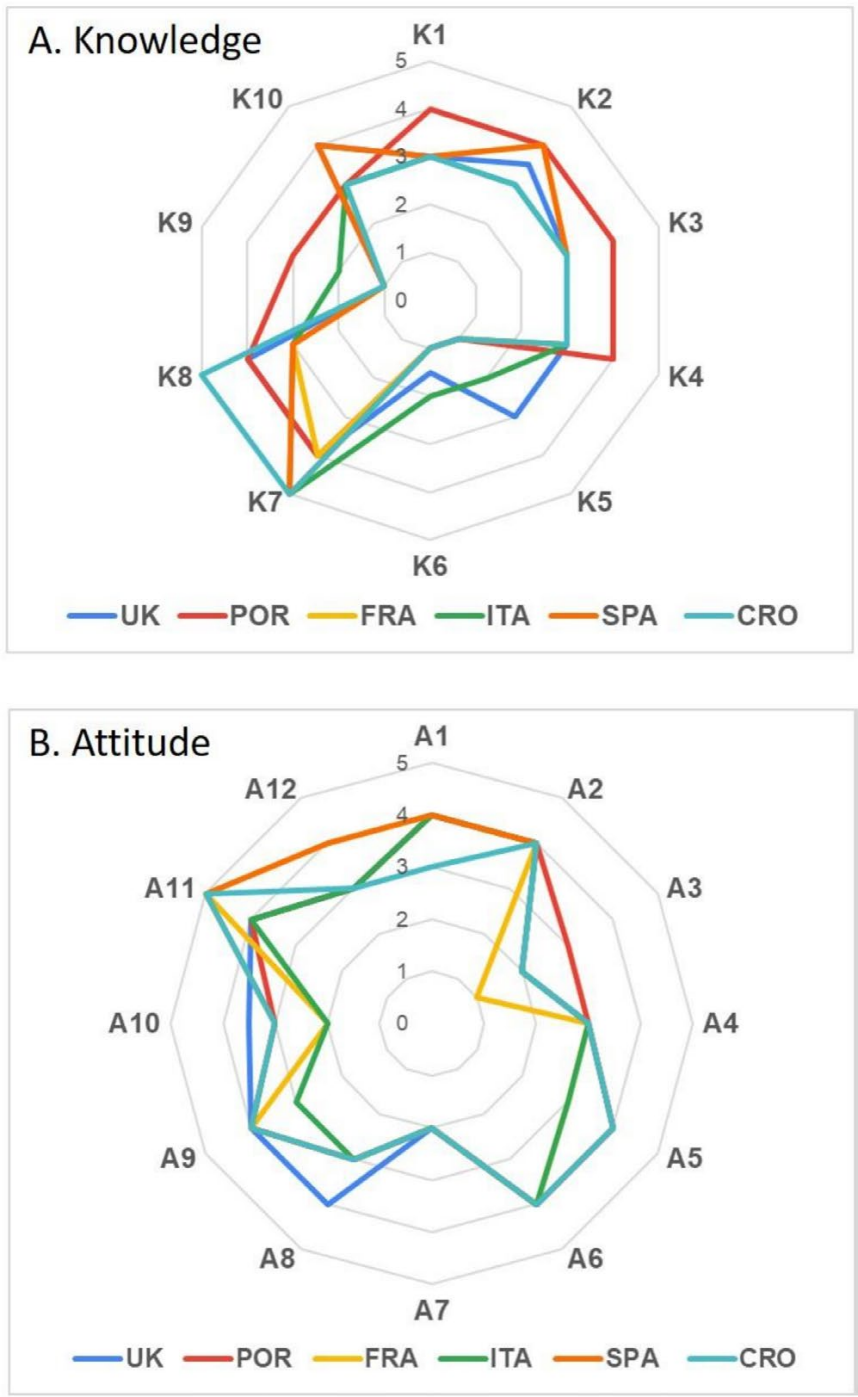
Variation in responses to individual statements assessing knowledge (A) and attitudes (B) towards the use of AI among students from different countries. (CRO, Croatia; FRA, France; ITA, Italy; POR, Portugal; SPA, Spain; UK, United Kingdom; K1‐10—statements assessing knowledge; A1‐12—statements assessing attitude). An almost non‐existent positive correlation (*r* = 0.171; *p* = 0.002) was observed between overall knowledge and overall attitude score.

Table 2 and Figure 1A present results on self‐perceived knowledge of AI. The highest proportion of the students (179; 54.6%) strongly disagreed with the statement “I have received formal training on the use of diagnostic‐aid AI tools – for example, those used in dental radiology, histopathology, and clinical dentistry”. The highest proportion of the students (154; 47%) strongly agreed with the statement “More training on the applications and use of AI is needed in undergraduate dental programmes”.

**TABLE 2:** Knowledge of artificial intelligence.

	Agreement *N* (%)					Difference by gender (*p*)	Difference by country (*p*)	Pairwise comparison. Post hoc Bonferroni correction *p*
	Strongly disagree 1	2	3	4	Strongly agree 5			
K1. I understand the basic computational principles of AI	21 (6.4)	63 (19.2)	100 (30.5)	92 (28)	52 (15.9)	M > F; 0.008*	< 0.0001*	POR>ITA; 0.005 POR>SPA; < 0.0001
K2. I understand the areas of my role as a dentist that may be modified by the introduction of AI	10 (3)	38 (11.6)	97 (29.6)	128 (39)	55 (16.8)	M > F; < 0.0001*	< 0.0001*	POR>ITA; 0.037 POR>CRO; 0.037 SPA>ITA; < 0.0001 SPA>CRO; < 0.0001
K3. I am comfortable with the terminology used to describe AI	23 (7)	58 (17.7)	113 (34.5)	88 (26.8)	46 (14)	M > F; < 0.0001*	< 0.0001*	POR>FRA; 0.018 POR>ITA; 0.001 POR>CRO; 0.006 FRA>CRO; 0.006 SPA>ITA; 0.027
K4. I have an understanding of the limitations of AI	18 (5.5)	47 (14.3)	105 (32)	106 (32.3)	52 (15.9)	M > F; 0.029*	0.004*	POR>ITA; 0.008
K5. I have received teaching on the use of AI tools in dentistry	179 (54.6)	62 (18.9)	55 (16.8)	26 (7.9)	6 (1.8)	0.584	0.001*	UK>POR; 0.003 UK>FRA; 0.008 UK>SPA; 0.007 UK>CRO; < 0.0001
K6. I have received formal training on the use of diagnostic‐aid AI tools—for example, those used in dental radiology, histopathology, and clinical dentistry	200 (61)	58 (17.7)	35 (10.7)	23 (7)	12 (3.7)	0.440	0.003*	ITA>CRO; 0.002 SPA>CRO; 0.045
K7. More training on the applications and use of AI is needed in undergraduate dental programmes^a^	8 (2.4)	15 (4.6)	53 (16.2)	98 (29.9)	154 (47)	0.247	0.007*	SPA>POR; 0.004
K8. I have first‐hand experience using AI tools – this may include text‐generative AI tools such as ChatGPT	26 (7.9)	41 (12.5)	75 (22.9)	79 (24.1)	107 (32.6)	0.101	0.002*	CRO>FRA; 0.030 CRO>SPA; 0.026
K9. I have first‐hand experience using AI tools within dentistry – for example, image analysis software	148 (45.1)	80 (24.4)	48 (14.6)	33 (10.1)	19 (5.8)	0.220	< 0.0001*	POR>FRA; < 0.0001 POR>SPA; < 0.0001 POR>CRO; 0.004 FRA>CRO; 0.004
K10. AI tools can be used to detect and diagnose oral potentially malignant disorders and oral cancers in a clinical setting	18 (5.5)	40 (12.2)	121 (36.9)	90 (27.4)	59 (18)	M > F; 0.011*	0.044*	ITA>POR; 0.018
Overall knowledge score (median [IQR))	28 (25–31)					M > F; < 0.0001*	0.001*	POR>FRA; 0.009 POR>CRO; 0.003

Abbreviations: CRO, Croatia; FRA, France; ITA, Italy; POR, Portugal; SPA, Spain; UK, United Kingdom.

^a^ Statement was reversely coded for the calculation of overall knowledge score.

* *p* < 0.05.

Male students expressed significantly higher agreement with the following statements: “I understand the basic computational principles of AI” (*p* = 0.008); “I understand the areas of my role as a dentist which may be modified by the introduction of AI” (*p* < 0.0001); “I am comfortable with the terminology used to describe AI” (*p* < 0.0001); and “AI tools can be used to detect and diagnose oral potentially malignant disorders and oral cancers in a clinical setting” (*p* = 0.011) compared to female students. Significant differences in agreement between students from different countries was observed for all statements. Figure 1A presents the variation in responses to individual statements among students from different countries. Details on differences in responses between students from different countries are presented in Table 2.

Table 3 and Figure 1B present the results on attitude towards AI and its applications in dentistry and oral medicine. The highest proportion of students (93; 28.4%) strongly disagreed with the statement “When considering a possible oral cancer or oral potentially malignant lesion, I would trust the assessment of an AI tool more than my own assessment”. The highest proportion of students (132; 40.2%) strongly agreed with the statement “I would be willing to test an AI based‐tool for diagnosis of oral cancers and oral potentially malignant disorders”.

**TABLE 3:** Attitudes towards artificial intelligence and its applications in dentistry.

	Agreement *N* (%)					Difference by gender (*p*)	Difference by country (*p*)	Pairwise comparison. Post hoc Bonferroni correction *p*
	Strongly disagree 1	2	3	4	Strongly agree 5			
A1. I think that AI will play an important role in the early diagnosis of potentially malignant disorders and oral cancers	9 (2.7)	35 (10.7)	97 (29.6)	112 (34.1)	75 (22.9)	M > F; 0.008*	< 0.0001*	CRO<UK; < 0.0001 CRO<POR; 0.043 CRO<FRA; 0.001 CRO<ITA; 0.026 CRO<SPA; < 0.0001
A2. I think that an AI tool to identify oral potentially malignant disorders and oral cancers, in combination with clinical examination, will benefit my patients	5 (1.5)	11 (3.4)	69 (21)	116 (35.4)	127 (38.7)	0.145	< 0.0001*	POR<FRA; 0.001 POR<SPA; < 0.0001
A3. I think that dentists will play a less important role in the diagnosis of oral potentially malignant disorders and oral cancer in the future due to AI^a^	119 (36.3)	87 (26.5)	75 (22.9)	29 (8.8)	18 (5.5)	0.564	0.013*	FRA<POR; 0.013 FRA<SPA; 0.020
A4. I think that some aspects of my role as a dentist will be replaced by AI within my lifetime	40 (12.2)	80 (24.4)	95 (29)	62 (18.9)	51 (15.5)	M > F; 0.027*	0.291	
A5. I think that the introduction of an AI tool to aid in the diagnosis of oral potentially malignant disorders and oral cancers will result in reduced incidence of clinical errors	11 (3.4)	26 (7.9)	114 (34.8)	117 (35.7)	60 (18.3)	M > F; 0.047*	0.679	
A6. I think that AI will improve the efficiency of diagnosis and timely management of oral potentially malignant disorders and oral cancers	4 (1.2)	28 (8.5)	96 (29.3)	124 (37.8)	76 (23.2)	M > F; 0.011*	0.448	
A7. When considering a possible oral cancer or oral potentially malignant lesion, I would trust the assessment of an AI tool more than my own assessment^§^	93 (28.4)	114 (34.8)	78 (23.8)	32 (9.8)	11 (3.4)	M > F; 0.005*	0.502	
A8. I think that an AI tool to aid the diagnosis of oral potentially malignant disorders and oral cancers will reduce the workload of clinicians	21 (6.4)	66 (20.1)	119 (36.3)	89 (27.1)	33 (10.1)	0.227	< 0.0001*	FRA<UK; 0.001 FRA<SPA; < 0.0001
A9. I worry that AI systems may be manipulated with bad intent, e.g., hackers, terrorism…^§^	24 (7.3)	29 (8.8)	80 (24.4)	96 (29.3)	99 (30.2)	0.211	0.003*	SPA<POR; 0.025 SPA<FRA; 0.025 ITA<POR; 0.004
A10. I think that the use of AI in dentistry will impair the patient‐dentist relationship^§^	56 (17.1)	85 (25.9)	88 (26.8)	64 (19.5)	35 (10.7)	0.097	< 0.0001*	FRA<UK; 0.020 FRA<POR; < 0.0001 SPA<POR; 0.007
A11. I would be willing to test an AI‐based tool for diagnosis of oral cancers and oral potentially malignant disorders	7 (2.1)	11 (3.4)	71 (21.6)	107 (32.6)	132 (40.2)	0.639	< 0.0001*	POR<FRA; < 0.0001 POR<SPA; < 0.0001 POR<CRO; 0.006 CRO<FRA; 0.006
	Very negative 1	2	3	4	Very positive 5	Difference by gender	Difference by country	
A12. Please rate how positive you feel about the use of AI in the diagnosis of oral cancers and oral potentially malignant disorders on the following scale	7 (2.1)	32 (9.8)	127 (38.7)	110 (33.5)	52 (15.9)	M > F; 0.002*	0.007*	POR<FRA; 0.025 POR<SPA; 0.013
Overall attitude score (median (IQR))	42 (38–46)					M > F; 0.01*	< 0.0001*	POR<FRA; 0.001* POR<SPA; < 0.0001*

Abbreviations: CRO, Croatia; FRA, France; ITA, Italy; POR, Portugal; SPA, Spain; UK, United Kingdom.

^a^ Statement was reversely coded for the calculation of overall attitude score.

* *p* < 0.05.

Male students expressed significantly higher agreement with the following statements: “I think that AI will play an important role in the early diagnosis of oral potentially malignant disorders and oral cancers” (*p* = 0.008), “I think that some aspects of my role as a dentist will be replaced by AI within my lifetime” (*p* = 0.027), “I think that the introduction of an AI tool to aid diagnosis of oral potentially malignant disorders and oral cancers will result in reduced incidence of clinical errors” (*p* = 0.047), “I think that AI will improve efficiency of diagnosis and improve timely management of oral potentially malignant disorders and oral cancers” (*p* = 0.011), “When considering a possible oral cancer or oral potentially malignant lesion, I would trust the assessment of an AI tool more than my own assessment” (*p* = 0.005) compared to female students. Male students also expressed more positive feelings about the use of AI in the diagnosis of oral cancers and oral potentially malignant disorders compared to female students (*p* = 0.002).

Significant differences among students from different countries was observed for the following statements: “I think that AI will play an important role in the early diagnosis of oral potentially malignant disorders and oral cancers” (*p* < 0.0001), “I think that an AI tool to identify oral potentially malignant disorders and oral cancers, in combination with clinical examination, will benefit my patients” (*p* < 0.0001), “I think that dentists will play a less important role in the diagnosis of oral potentially malignant disorders and oral cancer in the future due to AI” (*p* = 0.013), “I think that an AI tool to aid diagnosis of oral potentially malignant disorders and oral cancers will reduce the workload of clinicians” (*p* < 0.0001), “I worry that AI systems may be manipulated with bad intent e.g., hackers, terrorism…” (*p* = 0.003), “I think that the use of AI in dentistry will impair the patient‐dentist relationship” (*p* < 0.0001), “I would be willing to test an AI based‐tool for diagnosis of oral cancers and oral potentially malignant disorders” (*p* < 0.0001) and “Please rate how positive you feel about the use of AI in the diagnosis of oral cancers and oral potentially malignant disorders on the following scale” (*p* = 0.007). Figure 1B presents the variation in responses to individual statements among students from different countries. Details on differences in responses between students from different countries are presented in Table 3.

Table 4 presents potential barriers to the use of AI in the diagnosis of OPMDs and OC. The most reported perceived barrier was the lack of training in the use of such AI programmes (288; 87.8%). Fear of AI software replacing human clinicians was least commonly perceived barrier to AI use (173; 52.7%). Female students perceived more frequently the following barriers compared to male students: lack of an appropriately designed and tested AI software (84.1% vs. 67.5%; *p* = 0.004), lack of training in the use of such AI programmes (90.7% vs. 79.3%; *p* = 0.006), fear of AI software replacing human clinicians (58.1% vs. 36.6%; *p* = 0.001) and cost of AI software (79.7% vs. 61%; *p* = 0.001). Significant differences in the perception of the following barriers was found among students from different countries: lack of an appropriately designed and tested AI software (*p* = 0.030), lack of training in the use of such AI programmes (*p* = 0.001), fear of AI software replacing human clinicians (*p* < 0.001), fear of liability due to error made by AI (*p* = 0.015) and patient uncertainty about the use of AI tools in their care (*p* = 0.036).

**TABLE 4:** Perceived barriers to the use of AI in the diagnosis of oral potentially malignant disorders and oral cancer.

	Perceived as a barrier		Difference by gender (*p*)	Difference by country	Pairwise comparison. Post hoc Bonferroni correction *p* value
	Yes	No			
Lack of an appropriately designed and tested AI software	264 (80.5)	64 (19.5)	0.004*	0.030*	SPA>FRA; *p* = 0.0037*
Lack of awareness about the capabilities of AI	264 (80.5)	64 (19.5)	0.108	0.217	
Lack of training in the use of such AI programmes	288 (87.8)	40 (12.2)	0.006*	0.001*	SPA>POR; *p* = 0.0013*
Fear of AI software replacing human clinicians	173 (52.7)	155 (47.3)	0.001*	< 0.0001*	UK>ITA; *p* = 0.0019* POR>ITA; *p* = 0.0019*
Cost of AI software	246 (75)	82 (25)	0.001*	0.086	
Fear of liability due to an error made by AI	270 (82.3)	58 (17.7)	0.867	0.015*	SPA>CRO; *p* = 0.0027*
Fear of data misuse or manipulation by AI	270 (82.3)	58 (17.7)	0.403	0.086	
Patient uncertainty about the use of AI tools in their care	238 (72.6)	90 (27.4)	0.063	0.036*	Post hoc test did not detect significant difference

* *p* < 0.05.

**FIGURE 2:**
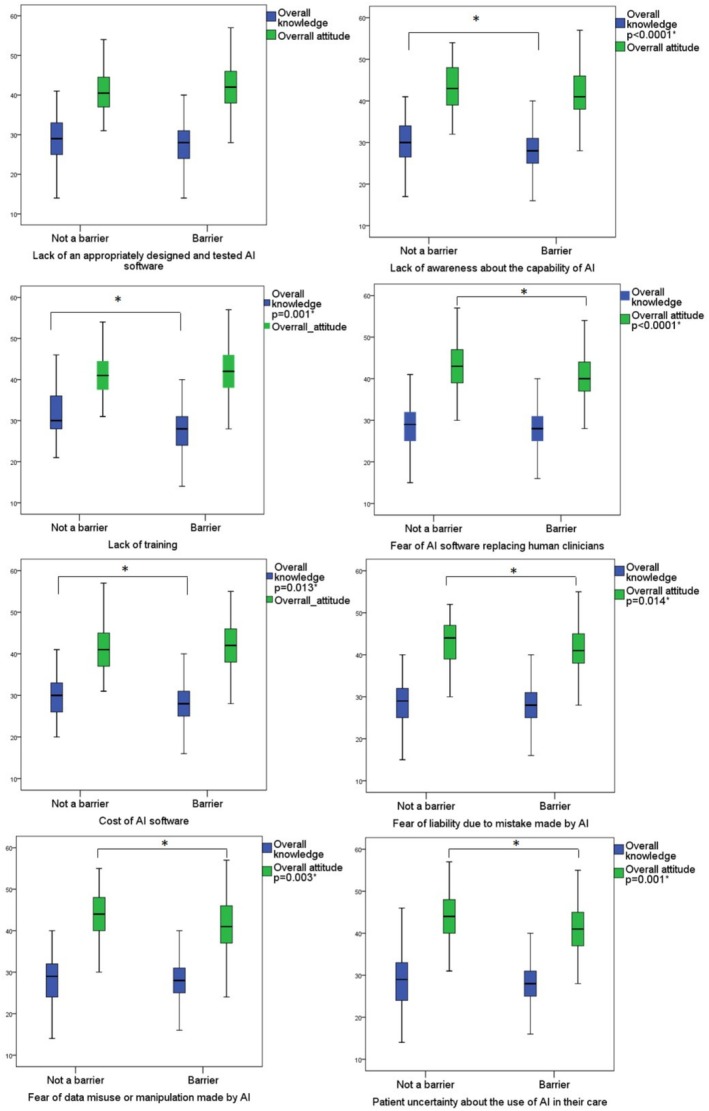
Overall knowledge and attitude scores in relation to the perception of barrier from the use of artificial intelligence‐based tools for the diagnosis of oral cancer and oral potentially malignant disorders.

The results showed that students who perceived the lack of awareness about the capability of AI, the lack of training in the use of AI programmes and the cost of AI software as obstacles to the use of AI in the diagnosis of OPMDs and OC had significantly lower overall knowledge score compared to the students who did not perceive those as an obstacle to the use of AI. We also observed that students who perceived the fear of AI software replacing human clinicians, the fear of liability due to error made by AI, the fear of data misuse or manipulation made by AI and the patient uncertainty about the use of AI tools in their care had significantly lower overall attitude score compared to the students who did not perceive those as an obstacle to the use of AI (Figure 2).

## 4 Discussion

This study demonstrates generally positive attitudes of final year European dental students towards the use of AI‐based tools to aid in the diagnosis of OC and OPMDs. In addition, the study identified important gaps in AI knowledge and training. This finding is in line with other studies reporting that both dentists and dental students often lack a comprehensive understanding of AI's potential within the profession (Amiri et al. 2024; Aldakhil et al. 2024). Although some studies suggest that students who report higher self‐perceived understanding of AI express more favourable attitudes towards its use, the correlation between overall knowledge and attitude in our study was very weak, almost non‐existent (Jeong et al. 2024; Yılmaz et al. 2024). This suggests that, while greater knowledge may contribute to more favourable attitudes, knowledge alone is unlikely to be sufficient to impact willingness to use AI in clinical practice. Factors such as perceived usefulness, trust in AI‐assisted decisions, ethical considerations and clinical exposure may therefore play an equal or more important role (Osman 2025; Qi et al. 2025).

Regarding gender differences, male students demonstrated higher knowledge and more positive attitudes towards the use of AI‐based tools to aid in the diagnosis of OC and OPMDs compared to female students. This difference may be explained by the consistently reported higher confidence in adopting and relying on digital technologies among male students (Compeau and Higgins 1995; Venkatesh and Morris 2000; Cai et al. 2017). Furthermore, Russo et al. (2025) reported that men exhibit lower levels of AI anxiety, greater usage, and more positive attitudes towards AI compared with women, factors that foster greater confidence and trust in AI‐based tools. Thus, male students' more positive attitude towards AI's diagnostic utility may stem from both lower affective barriers and higher confidence in one's ability to use such technologies effectively (Russo et al. 2025).

The differences observed in overall knowledge and attitudes across countries likely reflected variations in digital infrastructure, curricular emphasis on AI, institutional priorities, and the availability of formal AI‐related teaching. Similar cross‐country variability has been reported in studies assessing undergraduate education on OPMDs and oral biopsies, where differences in curriculum structure and clinical exposure/hands‐on experience were associated with differences in students' self‐perceived competence and confidence (Brailo et al. 2023, 2025).

There was also an association between low knowledge and perception of barriers: students with lower knowledge scores more frequently identified barriers to its use, including lack of awareness of the capability of AI, lack of training, and the cost of AI software. This finding is somewhat expected as insufficient education affects students' understanding of how AI functions, their diagnostic potential, and its limitations. On the other hand, students who perceived fear of AI replacing human clinicians, fear of liability due to errors made by AI, fear of data misuse or manipulation made by AI and patient uncertainty about the use of AI tools in their care had significantly lower attitude scores. These students were not less knowledgeable but tended to adopt a more cautious or sceptical perspective towards AI. Therefore, it is possible that their perceived barriers reflect ethical, professional and medico‐legal concerns rather than purely technical limitations. These barriers may arise when students are partially informed about AI, aware of its capabilities, but not adequately educated about aspects such as human oversight, data protection, or liability in AI‐assisted decision‐making. Lack of ethical and legal clarity surrounding clinical applications of AI might contribute to mistrust and negative attitudes, even when basic technical knowledge about AI exists (Feng et al. 2025; Zhang and Zhang 2023; Berg 2004).

The study has several limitations that need to be mentioned. The sample size was limited to students from eight universities across six European countries, and its results cannot be generalised to all European dental students. Non‐response bias, i.e., the risk that students who chose not to participate may differ in their responses from those who did, should also be acknowledged. Furthermore, uneven sample sizes across participating countries and the predominance of female respondents may have influenced the observed gender and regional differences. Finally, the cross‐sectional design of the study registered students' attitudes and perceptions at a single time point, preventing conclusions about changes over time. The rapid evolution of AI tools in dentistry implies that students' knowledge and perceptions may shift quickly, limiting the long‐term generalisability of these findings.

## 5 Conclusion

Whilst undergraduate dental students across Europe generally hold positive attitudes towards the use of AI in diagnosing oral OC and OPMDs, there remains a notable lack of knowledge and formal training and structured education in this area. Gender and country‐specific differences suggest that technological self‐efficacy and exposure may play an important role in shaping students' perceptions and trust in AI‐assisted diagnostics. The most significant barrier identified was insufficient training, underscoring the need to integrate education about technical as well as ethical and legal aspects of AI use into undergraduate curricula. A meaningful step forward would be to develop a more coordinated approach to AI education at the European level. This could be achieved through collaboration among dental schools, expert groups and professional organisations already active in oral medicine and dental education. Activities should be focused on defining core learning outcomes (e.g., understanding of basic computational principles and limitations of AI, data ethics, and safe clinical application), developing open‐source teaching materials, and promoting inter‐institutional teaching collaborations. Furthermore, incorporating case‐based simulations and supervised exposure to AI‐supported diagnostic tools could help bridge the gap between theoretical knowledge and clinical application. Such an initiative would not only reduce disparities among European universities but also ensure that future dental professionals across Europe are equally prepared to critically evaluate and responsibly use AI in diagnosing OC and OPMDs. Interventions that emphasise the use of AI as an adjunctive, not substitutive clinical tool may help to reduce apprehension and promote professional trust.

## Author Contributions

**Vlaho Brailo:** data curation, formal analysis, investigation, writing – original draft preparation. **Rosa María Lopez‐Pintor:** formal analysis, investigation, writing – original draft preparation. **Molly Harte:** data curation, methodology, investigation, resources, writing – original draft preparation, visualisation. **Ana Andabak Rogulj:** formal analysis, investigation. **Danica Vidović Juras:** data curation, formal analysis, investigation, writing – original draft preparation. **Ivana Škrinjar:** formal analysis, investigation. **Marcio Diniz Freitas:** data curation. **Jacobo Limeres Posse:** data curation. **Luis Monteiro:** data curation. **Luis M. Silva:** data curation. **Jean‐Cristophe Fricain:** data curation. **Adrien Naveau:** data curation. **Giovanni Lodi:** data curation. **Niccolò Lombardi:** data curation. **Elena Varoni:** data curation. **José López López:** data curation. **Sonia Egido Moreno:** data curation. **Raj Ariyaratnam:** visualisation, writing – review and editing. **Ali Abdullah Alqarni:** writing – review and editing. **Richeal Ní Ríordáin:** investigation, methodology, data curation. **Owen Addison:** writing – review and editing. **Yunpeng Li:** writing – review and editing. **Faleh Tamimi:** writing – review and editing. **Rui Albuquerque:** conceputalization, methodology, supervision, visualization, writing – review and editing.

## Funding

This research has been funded by the Instituto de Salud Carlos III (ISCIII) through the project PI22/00905, co‐funded by the European Union.

## Ethics Statement

Ethical approval for the study was granted by the Ethics committees of all universities participating in the study.

## Conflicts of Interest

The authors declare no conflicts of interest.

## Data Availability

The data that support the findings of this study are available in Appendix A of this article.

## Appendix A Questionnaire Distributed to Participants

**Demographic data**

1. In which country do you study dentistry?
  England
  Ireland
  Portugal
  France
  Italy
  Spain
  Croatia
2. What is your gender?
  Male
  Female
  Non‐binary
  Other
  Prefer not to say
3. What is your age?
4. Do you have any prior degree‐level qualifications?
  Yes, please specify:
  No

**Questionnaire**

Please indicate to what extent you agree/disagree with the following statements:

**Table:**

	Strongly disagree	Disagree	Neutral	Agree	Strongly agree
**Knowledge of artificial intelligence**					
I understand the basic computational principles of AI					
2 I understand the areas of my role as a dentist that may be modified by the introduction of AI					
3 I am comfortable with the terminology used to describe AI					
4 I have an understanding of the limitations of AI					
5 I have received teaching on the use of AI tools in dentistry					
6 I have received formal training on the use of diagnostic‐aid AI tools – for example, those used in dental radiology, histopathology, and clinical dentistry					
7 More training on the applications and use of AI is needed in undergraduate dental programmes					
8 I have first‐hand experience using AI tools – this may include text‐generative AI tools such as ChatGPT					
9 I have first‐hand experience using AI tools within dentistry – for example, image analysis software					
10 AI tools can be used to detect and diagnose potentially malignant oral disorders and oral cancers in a clinical setting					
**Attitudes towards artificial intelligence – as a clinician**					
I think that AI will play an important role in the early diagnosis of oral potentially malignant disorders and oral cancers					
2 I think that an AI tool to identify oral potentially malignant disorders and oral cancers, in combination with clinical examination, will benefit my patients					
3 I think that dentists will play a less important role in the diagnosis of oral potentially malignant disorders and oral cancer in the future due to AI					
4 I think that some aspects of my role as a dentist will be replaced by AI within my lifetime					
5 I think that the introduction of an AI tool to aid in the diagnosis of oral potentially malignant disorders and oral cancers will result in reduced incidence of clinical errors					
6 I think that AI will improve the efficiency of diagnosis and timely management of oral potentially malignant disorders and oral cancers					
7 When considering a possible oral cancer or oral potentially malignant lesion, I would trust the assessment of an AI tool more than my own assessment					
8 I think that an AI tool to aid the diagnosis of oral potentially malignant disorders and oral cancers will reduce the workload of clinicians					
9 I worry that AI systems may be manipulated with bad intent e.g., hackers, terrorism					
10 I think that the use of AI in dentistry will impair the patient‐dentist relationship					
11 I would be willing to test an AI‐based tool for the diagnosis of oral cancers and oral potentially malignant disorders					

**Barriers to the use of AI in the diagnosis of oral potentially malignant disorders and oral cancers**

Please indicate which of the following you perceive to be barriers to the use of AI in the diagnosis of oral potentially malignant disorders and oral cancers.

**Table:**

	Perceived barrier	Not a barrier
Lack of an appropriately designed and tested AI software		
Lack of awareness about the capabilities of AI		
Lack of training in the use of such AI programmes		
Fear of AI software replacing human clinicians		
Cost of AI software		
Fear of liability due to an error made by AI		
Fear of data misuse or manipulation by AI		
Patient uncertainty about the use of AI tools in their care		

**Global assessment**

Please rate how positive you feel about the use of AI in the diagnosis of oral cancers and oral potentially malignant disorders on the following scale:

**Table:**

Very negative	Negative	Neutral	Positive	Very positive
